# The Role of Genistein in Mammalian Reproduction

**DOI:** 10.3390/molecules28217436

**Published:** 2023-11-05

**Authors:** Gabriella Guelfi, Rolando Pasquariello, Polina Anipchenko, Camilla Capaccia, Georgia Pennarossa, Tiziana A. L. Brevini, Fulvio Gandolfi, Massimo Zerani, Margherita Maranesi

**Affiliations:** 1 Department of Veterinary Medicine, University of Perugia, 06126 Perugia, Italy; gabriella.guelfi@unipg.it (G.G.); camilla.capaccia@gmail.com (C.C.); massimo.zerani@unipg.it (M.Z.); margherita.maranesi@unipg.it (M.M.); 2 Department of Agricultural and Environmental Sciences, University of Milan, 20133 Milano, Italy; rolando.pasquariello@unimi.it (R.P.); fulvio.gandolfi@unimi.it (F.G.); 3 Department of Veterinary Medicine and Animal Science, University of Milan, 26900 Lodi, Italy; tiziana.brevini@unimi.it

**Keywords:** polyphenols, flavonoids, isoflavones, genistein, phytoestrogens, ovary, testis, pregnancy

## Abstract

Genistein is a natural compound belonging to flavonoids, having antioxidant, anti-inflammatory, and anti-neoplastic properties. Genistein is considered a phytoestrogen. As such, genistein can bind estrogen receptors (ERα and ERβ), although with a lower affinity than that of estradiol. Despite considerable work, the effects of genistein are not well established yet. This review aims to clarify the role of genistein on female and male reproductive functions in mammals. In females, at a high dose, genistein diminishes the ovarian activity regulating several pathway molecules, such as topoisomerase isoform I and II, protein tyrosine kinases (v-src, Mek-4, ABL, PKC, Syk, EGFR, FGFR), ABC, CFTR, Glut1, Glut4, 5α-reductase, PPAR-γ, mitogen-activated protein kinase A, protein histidine kinase, and recently circulating RNA-miRNA. The effect of genistein on pregnancy is still controversial. In males, genistein exerts an estrogenic effect by inducing testosterone biosynthesis. The interaction of genistein with both natural and synthetic endocrine disruptors has a negative effect on testis function. The positive effect of genistein on sperm quality is still in debate. In conclusion, genistein has a potentially beneficial effect on the mechanisms regulating the reproduction of females and males. However, this is dependent on the dose, the species, the route, and the time of administration.

## 1. Characteristics and Beneficial Effects of Polyphenols

In the vegetable kingdom, polyphenols are found in abundance in the edible parts of plants. Moreover, they are fundamental compounds in animal and plant physiology [1,2]. Polyphenols contribute to plant and flower pigmentation, and their aroma attracts pollinator insects and confers the resistance of the plant to microorganisms and stress (i.e., UV rays, etc.) [3]. Furthermore, it should be emphasized that the polyphenol contents of edible parts of plants are of fundamental importance for the food and pharmaceutical industry due to their health-enhancing and disease-preventing properties [4,5]. Polyphenols include more than 8000 compounds characterized by the presence of multiple phenolic groups associated with complex structures of high molecular weight [6].

Polyphenols are compounds with a similar chemical structure derived from benzene and one or more ring-associated hydroxyl groups. Based on the number of aromatic rings and the elements that bind these rings, polyphenols are classified into distinct groups and subgroups correlated with their peculiarities in terms of function and benefits. Various beneficial properties are generally attributed to all polyphenols [7]: antioxidant [8], immunomodulatory, anti-inflammatory [9], cardioprotective [10], neuroprotective, antimicrobial, hepatoprotective, anti-platelet, anti-tumor, anti-diabetic, anti-obesity, anti-osteoporosis, and anti-estrogenic as estrogen receptor agonists. In the veterinary field, polyphenols are known as anti-parasitic [11] and anti-aging molecules [12]. Moreover, the anti-bacterial and anti-mycotic activity of polyphenols has been widely demonstrated [13,14]; this is relevant in livestock farming, as it helps reduce the use of antibiotic and antimycotic drugs.

**Figure 1:**
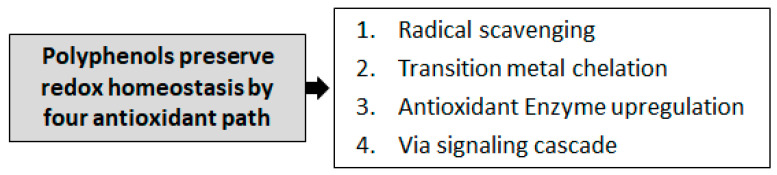
Four main mechanisms underlying the antioxidant activity of polyphenols.

Phenolic compounds mainly protect against systemic and/or local inflammation by reducing oxidative stress and regulating cytokine-mediated inflammatory responses [15]. Critical mechanisms of reactive oxygen species (ROS) production usually include psycho-physical stress, eating disorders, environmental pollution, and many pharmacological substances. If homeostatic mechanisms are not regained, prolonged production of ROS can trigger degenerative pathways leading to chronic pathogenic changes, including cancers and non-physiological aging. On the other hand, the reduction in excessive ROS production improves the antioxidant barrier and helps prevent and fight multiple diseases. Polyphenols can help protect against ROS damage by inhibiting the oxidation of substrates such as phospholipids, proteins, and DNA [16]. However, evidence that ROS are also physiologically essential cannot be ignored; ROS take part in different cellular pathways [17,18]. The prevailing theory is that polyphenols neutralize free radicals by forming stable chemical complexes. Polyphenols act through four main mechanisms to maintain redox homeostasis: radical scavenging, transition metal chelation, antioxidant enzyme upregulation, and signaling cascades (Figure 1). 

In addition to these four proposed mechanisms, polyphenols can prevent oxidative stress by producing hydrogen peroxide, a strategy that helps regulate immunological responses and cell growth. Following the theory of radical scavenging, the structure of polyphenols yields hydrogen atoms to free radical species, thus reducing their concentration and the risk they pose [19,20]. ROS are the result of physiological chemical reactions involving oxygen. ROS comprise superoxide (O^2−^), the hydroxyl radical (OH), and hydrogen peroxide (H_2_O_2_) [21]. ROS are highly reactive molecules that contain at least one unpaired electron in their outermost orbital. ROS are highly unstable and try to return to their equilibrium state by subtracting an electron needed to balance their electromagnetic charge from other adjacent atoms [22].

This process produces further new unstable molecules, triggering a chain reaction that can damage cellular structures if not kept within physiological values. Mitochondrial cellular respiration, essential for ATP production, is the main producer of ROS. Therefore, ROS production significantly increases during intense muscular activity. 

The transition metal chelation theory argues that polyphenols bind free metals, particularly iron, inside cells, thereby preventing the Fenton reaction by which free iron reacts with hydrogen peroxide to produce hydroxyl radicals [23]. However, under physiological conditions, the iron concentration is perfectly regulated by intracellular enzymes. ‘Radical scavenging’ and ‘transition metal chelation’ are the most widely reported mechanisms of action of polyphenols *in vitro* and *in vivo* [24].

Polyphenols activate genes involved in pathways inducing the upregulation of antioxidant/detoxifying enzymes [25]. The Keap1/Nrf2/ARE pathway firstly induces the dissociation of Keap1 (Kelch-like ECH-associating protein 1). After that, Nrf2 (nuclear factor erythroid 2 related factor 2) translocates into the nucleus, where it binds the regulatory region of the ARE (antioxidant response element) determining the transcription of antioxidant/detoxifying enzymes [26].

The redox homeostasis preserved via the signaling cascade is a mechanism involving mainly protein kinase C (PKC) [26]. PKC is implicated in the phosphorylation of ubiquitinated proteins that directly or indirectly control the activation or inhibition of numerous cellular pathways, such as apoptosis, cell proliferation, transcription regulation, immune responses, and cell signaling [27]. After phosphorylation, PKCs switch to the active conformational state and can be involved in cellular communication mechanisms by phosphorylating target proteins that regulate apoptotic or anti-apoptotic pathways [28]. The phosphorylation of Nrf2 by PKC determines the subsequent regulation of antioxidant enzymes; this is one of the mechanisms used by polyphenols to preserve the redox homeostasis of the cell via PKC [26]. 

## 2. Biotransformation of Polyphenols and Physiological Responses 

To evaluate the biological effect of a specific polyphenol, its absorption, bioavailability, bioaccessibility, and bioactivity must all be considered. The absorption, metabolism, and bioavailability of polyphenols are dependent on the type of polyphenol provided in one’s diet. 

Absorption is described as the path of the bioactive compound from the point of administration to the site of action [29,30]. The absorption of polyphenol is influenced by the physicochemical properties of the polyphenol. The transport of a polyphenol across the cell membrane can be via passive diffusion, facilitated passive diffusion, active transport, or pinocytosis. Polyphenol absorption is well described in the literature; however, several doubts remain about polyphenol catabolism performed by gut microbiota [31]. In some cases, the gut microbiome may produce a metabolite level of ingested polyphenols that exceeds the quantity consumed. The metabolism of a polyphenol generally has the function of inactivating and eliminating it, although some metabolites are sometimes pharmacologically more active than the parent substance. The enzymes involved in metabolism are present in many tissues but generally are more concentrated in the liver. The metabolism is performed in two distinct phases. Phase I reactions, which are non-synthesis reactions, involve the formation of a new or modified functional group or cleavage (oxidation, reduction, or hydrolysis). Phase II reactions, which are synthetic reactions, involve conjugation with an endogenous substance (i.e., glucuronic acid, sulfate, or glycine) [1]. 

According to its nutritional definition, bioavailability is the fraction (%) of bioactive compounds that are distributed in the bloodstream and available or stored by an organism. Before becoming bioavailable, bioactive compounds must be released from the feed matrix and modified in the gastrointestinal tract; they are believed to be bioavailable compounds only after this point. Bioavailability includes absorption, intestinal and hepatic epithelia, digestive biotransformation, tissue distribution, and bioactivity [32,33]. Bioavailability is affected by the rapidity with which these nutrients are adsorbed and available at the action site. The critical issue is that bioavailability may be reduced significantly by low intestinal absorption, elevated metabolism, or both. Furthermore, in blood and target organs, polyphenol metabolites after enzymatic digestion may differ from native substances in terms of biological activity [34]. To investigate polyphenol bioavailability, the most widespread strategy involves the *in vivo* assumption of a feed containing the tested polyphenol. Therefore, the transitory increased blood concentration will mainly reflect the ability of a organism to absorb the polyphenols from the feed [33]. 

**Figure 2:**
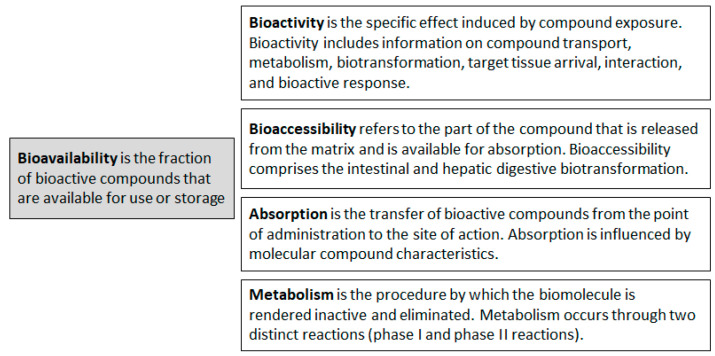
The bioavailability of polyphenols in an organism is determined by bioactivity, bioaccessibility, absorption, and metabolism.

Bioaccessibility indicates the part of the compound that is released from the food matrix and available for absorption; this happens, for example, when the compound enters the bloodstream. Bioaccessibility includes the intestinal and hepatic epithelia’s digestive biotransformations [35]. Bioaccessibility is extremely reliant on the molecular properties of the polyphenol (chemical structure [36] and interaction with other compounds [37,38]), food-related factors (food processing procedure [39], and food interacting with matrix effectors of positive or negative absorption [40]), host-related factors (absorption, metabolism, and host gut microbiota [41]), and other factors (related to the cultivation or plant growth environment [42,43]). Bioactivity is the specific effect induced by exposure to a bioactive compound. Bioactivity includes information on how bioactive compounds are transported, how they arrive at the target tissue, how they interact with biomolecules, their metabolism characteristics, biotransformation, and consequent physiological responses (Figure 2). 

**Figure 3:**
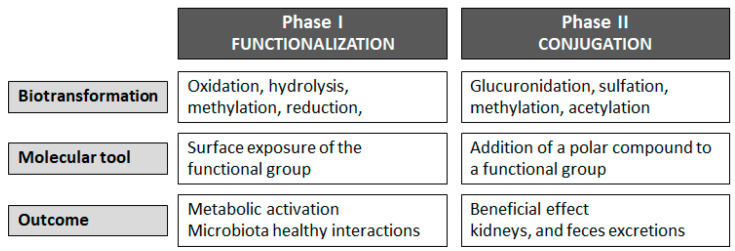
Phase I and phase II biotransformation of dietary polyphenols.

Dietary phenolic biotransformation begins in the oral cavity through metabolic reactions. Most polyphenols are present in foods in the form of esters, glycosides, or polymers, which are compounds that the organism metabolizes as extraneous to its normal nutrition, namely xenobiotic compounds (derived from a Greek expression meaning “foreign to bio”). Therefore, polyphenols must be biotransformed [44]. As opposed to the corresponding aglycones (the non-sugar part), the glycosides of polyphenols (the sugar part) must be biotransformed because they cannot cross the membrane by passive diffusion. In contrast, aglycones cross the epithelial cell membrane by passive diffusion. Biotransformation reactions occur to increase polyphenol hydrosolubility by preventing its accumulation and facilitating its elimination [45]. Therefore, in the oral cavity, only the metabolism of glycosylated phenolic compounds begins with oral microflora enzymes. In the gastrointestinal tract, polyphenolics are subjected to phase I or functionalization reactions and phase II or biotransformation reactions as they need some structural modifications for polyphenol absorption. It is to be emphasized that the biotransformation phase of polyphenols varies depending on the specific type of polyphenol [46]. Phase I concerns functionalization, i.e., the introduction or exposure of functional groups to the compound chemical structure. Phase I includes oxidation, methylation, reduction, and hydrolysis reactions. In phase II, the functional group of the metabolite derived from phase I is conjugated with endogenous molecules such as glucuronic acid, sulfuric acid, and methyl group. The primary aim of Phase II is to improve the polarity (hydrosolubility) of compounds from phase I, facilitating their elimination [46]. Phase I and II biotransformations can increase, decrease, or counteract the biological activity of phenolic compounds (Figure 3).

Polyphenols derived from gastric and intestinal absorption and microbial metabolism are transferred to the liver via enterohepatic circulation and in the liver, undergo phase I and II biotransformation. Regarding the biotransformation reaction, the liver is the primary organ where phenolic compounds are metabolized. Polyphenol metabolites, derived from hepatic metabolism, transfer to the bloodstream to be released through peripheral tissues to exert beneficial metabolic effects. Unabsorbed polyphenols and metabolites are excreted in urine and feces [47].

An emerging interest in the polyphenol domain is their potential interactions with gut microbiota, speculating that polyphenol metabolites may foster healthy gut bacteria while inhibiting invading species (a prebiotic effect) [48,49]. Upon the completion of the biotransformation process, the polyphenol is ready to be absorbed by organs and tissues to exert its effect or to be excreted via the most common ways, that is, via the kidneys, bile, or feces.

## 3. The Largest Group of Natural Polyphenols: Flavonoids

**Figure 4:**
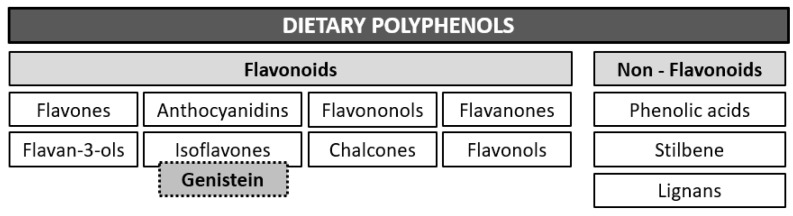
General classification of dietary polyphenols. Polyphenols may be divided into two main classes, flavonoids (comprising eight subclasses) and non-flavonoids (three subclasses).

Depending on the number of phenolic rings contained in dietary polyphenols, they can be classified into four subclasses as follows: phenolic acids which derivate from hydroxybenzoic acids and hydroxycinnamic acid; stilbenes; lignans; and flavonoids [50]. Flavonoid compounds are subdivided into flavones, flavonols, flavanones, isoflavones, anthocyanidins, flavan-3-ols, flavononols, and chalcones (Figure 4).

**Figure 5:**
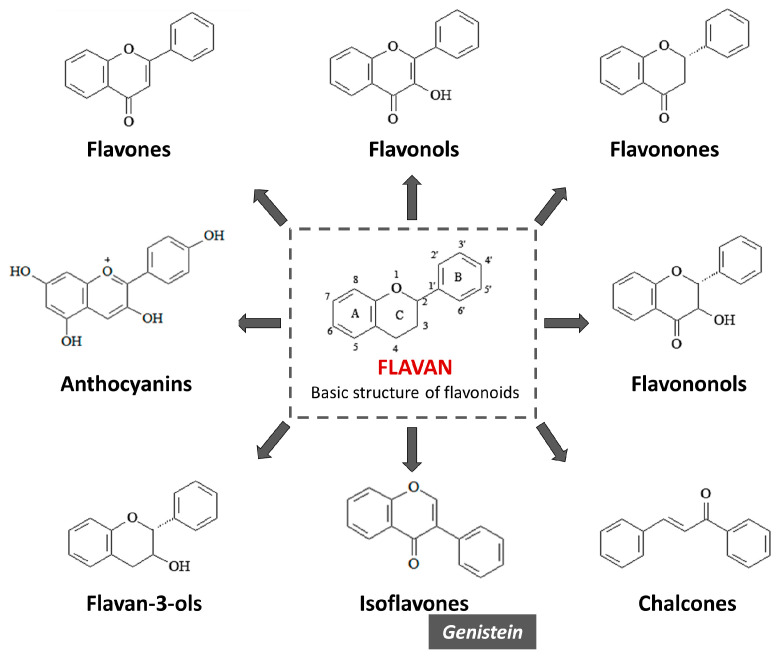
The flavonoid structure. Flavan is the basic skeleton structure of flavonoids and their classes.

Flavonoids constitute a category of polyfunctional substances with high bioactivity, which includes more than 5000 compounds. The word flavonoid comes from ‘flavus’ (=blond) and refers to its role in the pigmentation of plants. The color that flavonoids give a plant depends on the pH of the plant tissue. Blue pigments are formed by chelation with some metal ions. Anthocyanins, a specific group of flavonoids, are responsible for the aroma and color of flowers and fruit and therefore play an important role as mediators of pollination. It is important to observe that flavonoids, flavones, and flavonols, while not colorful to the human eye, highly absorb UV spectra and thus can be seen by insects [51]. While flavonoids are very visible in flower petals, in leaves, they are completely hidden by the ubiquitous green of chlorophylls. However, there is increasing evidence that these flavonoids, located on the upper surface of the leaf or in the epidermal cells, are crucial for plant protection against UV-B rays [52]. The most striking evidence of the role of flavonoids in UV-B protection is provided by *Arabidopsis thaliana*; mutants deprived of the epidermal flavonoids of this wild plant are highly susceptible to UV-B radiation [53]. Flavonoids constitute the group of natural phenols having flavan as their basic structure. The flavan compound is formed of two benzene rings (the A and B ring) attached by a pyran ring, i.e., a compound with three carbon atoms and an oxygen atom (C ring), and closed with the benzene ring A. The flavonoid structure is defined as C6–C3–C6. Generally, the B ring is bound to the C ring at position 2, but it can also be bound at position 3 or 4. The multiple possibilities for ring B’s binding mean that flavonoids constitute the most diversified group of polyphenols present in nature. Flavonoids in which the B ring binds to the C ring at position 3 are isoflavones [54]. In most cases, the B ring binds to position 2 of the C ring, which is the case for the following six subgroups based on the structural characteristics of the C ring: flavones, flavonols, flavonones, anthocyanins, flavononols, and flavan-3-ols (Figure 5). 

**Figure 6:**
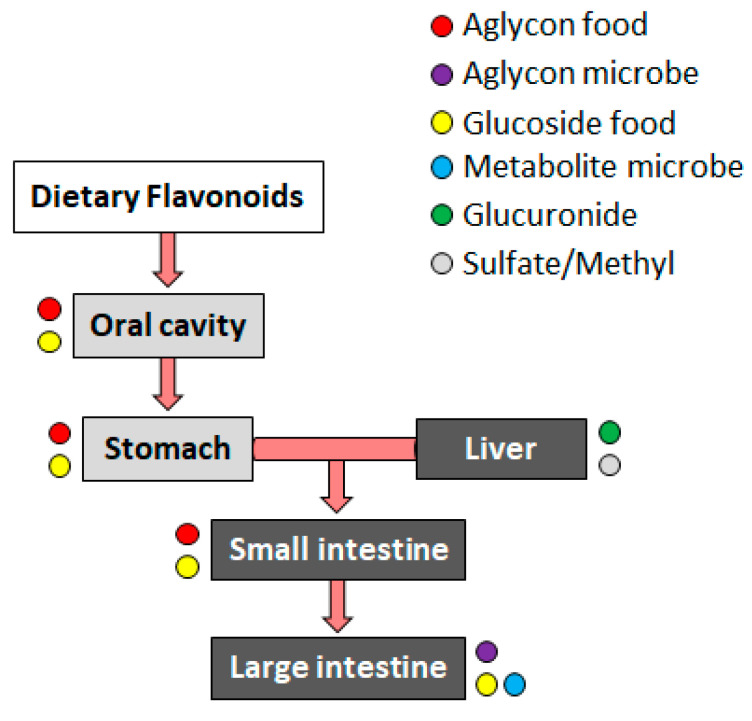
Schematic representation of dietary flavonoid metabolism. In the oral cavity, flavonoid aglycones and glucosides are metabolized by the oral microbiome. In the liver, these compounds are subjected to oxidative and reductive modifications that produce sulfites, methyls, and glucuronides. Mainly aglycones and flavonoid glucosides are present in the stomach and small intestine. Phase II metabolites derived from the digestion of the intestinal microbiota are also found in the large intestine.

A high number of studies have demonstrated that flavonoids are metabolized and bioactivated already in the oral phase of digestion through oral microbiome β-glucosidase activity, producing health effects in the oral cavity [55,56]. Flavonoid aglycones are more bioavailable than in the glycosylated form because they are absorbed by passive diffusion through the intestinal epithelium [57]. Flavonoid aglycones with flavonoid glucoside are present in the oral cavity, stomach, and small and large intestine. The hydroxyl groups of flavonoid aglycones or flavonoid metabolites of phase I metabolism are targets for phase II enzymes in both the liver and small intestine (Figure 6). 

In the intestine, flavonoids inhibit the activity of glucose-dependent insulinotropic polypeptide (GIP), an incretin family hormone released after a meal to decrease blood sugar [58,59]. In the small intestine, flavonoid glucuronidation mediated by intestinal cells plays a relevant role in flavonoid bioavailability. In the liver and small intestine, flavonoid aglycones undergo NADPH-dependent cytochrome P450 oxidative and reductive modifications [57]. Phase II enzymes have an essential role in flavonoid distribution and bioavailability as they are involved in various flavonoid recycling modes (enteric, enterohepatic, and cell-specific) [60,61,62]. It is important to note that flavonoid bioavailability is influenced by flavonoid molecule-related factors, such as the intake rate, chemical characteristics, and dietary matrix, and host-related factors, such as sex, age, gut microbiome, nutritional condition, and physiological state. In the last decade, the prominent role of the brain–gut axis in the microbiome’s interaction with flavonoid metabolism has emerged, mainly in terms of the immune response [63,64,65,66]. The intestinal microbiome can deglycosylate simple and complex flavonoid glycosides into their corresponding aglycones and convert aglycones, by C ring bacterial disruption, into new phenolic metabolites [67]. It has been demonstrated that flavonoids influence gut microbiome composition and diversity by inhibiting not only the growth of pathogenic bacteria but also the enhancement of commensal bacterial [68].

**Figure 7:**
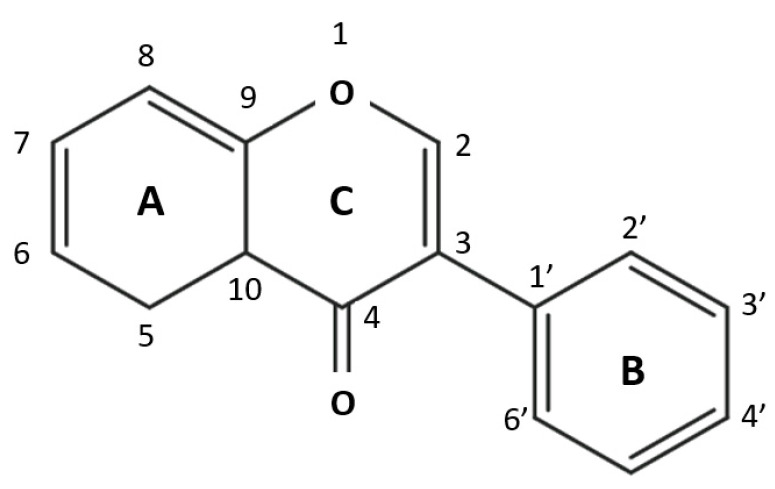
Chemical structure. Isoflavone ring connection and carbon numbering [69].

For structural and functional reasons, isoflavones have historically been regarded as a separate category within flavonoids. Isoflavones have an aromatic substituent at carbon C-3 due to the branching action of isoflavone synthase (Figure 7). 

Isoflavones include genistein, which has many of the biological properties of the flavonoid family; antioxidant properties have been demonstrated in genistein [69]. 

In contrast with other flavonoids, isoflavones have an estrogenic action. This property was observed in 1940 when Australian ewes feeding on subterranean clover (*Trifolium subterraneum* L.) showed fertility dysfunctions. Subsequent research on regional cattle forage revealed the estrogenic action of isoflavones, such as formononetin, daidzein, and its metabolite—equol—found in popular clover varieties (*Trifolium pretense* L. and *Trifolium repens* L.), which were also linked to reproductive issues in other vertebrates [70,71].

On the subject of “polyphenols and reproduction”, there are exhaustive and interesting reviews (e.g., polyphenols and ruminant reproduction [72], endometriosis [73], ovarian failure [74], and pregnancy [75]). Several other reviews have studied the specifics of isoflavones as phytoestrogens and their biological functions in depth [76,77,78,79,80,81]. However, this is the first review describing the role of isoflavones in reproduction, paying special attention to genistein in animals of veterinary interest. In this review, our current knowledge on genistein’s effect on female and male reproduction will be summarized with a particular emphasis on its mechanism of action.

## 4. Genistein and Reproduction

The use of nutraceuticals such as polyphenols and especially flavonoids in the reproductive field undoubtedly has utility in animals and humans [75,82].

The need to increase the reproductive efficiency of animals, avoiding the use of synthetic compounds while increasing animal welfare, has placed these natural compounds in the foreground as molecules to be added to animal feed thanks to their countless beneficial effects [72]. Among the flavonoids, soy isoflavones, particularly genistein, are of the greatest interest because of the widespread human and animal consumption of soy. In fact, soy products are the most common dietary source of isoflavones [83].

In addition to being high in protein, soybeans provide therapeutic nutrition due to their wide range of hormonal and non-hormonal properties. Possible health anticarcinogenic [84], antiangiogenic [85], and antioxidative-preventing osteoporosis benefits [86] have raised recent interest.

The actions of soy isoflavones are structurally related. They are nonsteroidal heterocyclic phenols with structural similarities to estradiol 17β and selective estrogen receptor modulators, and they exhibit significant estrogenic and anti-estrogenic activity. Among the key phytochemicals in soybean, phytoestrogens, specifically the isoflavones genistein, daidzein, and glycitein, have received the greatest attention.

According to a study on soy intake in various countries, the average daily soy intake in Asian countries is nine times higher than in North American and European countries, contributing to a better life expectancy on average [87].

In general, soy food has the highest quantities of genistein and daidzein and a wide range of content. An interesting review by Jefferson (2010) [88] reports the content of daidzen and genistein in some foods. Cereals containing soy, for example, contain 10–40 mg of daidzein and genistein/100 g. Meatless dishes have greater levels of phytoestrogens; there is 64 mg of daidzein as well as 46 mg of genistein in meatless bacon pieces per 100 g. Traditional Asian dishes such as miso have 16 mg daidzein and 23 mg genistein per 100 g. Tofu milk contains 28 mg daidzein and 43 mg genistein per 100 g [88].

Although there is much evidence of the beneficial effect of flavonoids, there are also studies stating that their use has controversial effects [89]. A recent review by Duda-Chodak (2023) [90] highlights adverse health effects from the use of polyphenols and flavonoids especially when used as supplements, also analyzing their potential mutagenic, genotoxic, neoplastic effects and interference with hormonal, digestive, and drug interactions.

## 5. Genistein’s Effect on Females

Despite several studies, it remains unclear if genistein can be utilized alone or in combination with other estrogenic compounds to modulate animal reproductive function or in estrogen replacement treatment. In the following paragraphs, the main effects of genistein as a phytoestrogen, in the ovary and during pregnancy, will be discussed.

### 5.1. Genistein as a Phytoestrogen

The term “phytoestrogenicity” refers to the estrogenic-like activity of some naturally derived polyphenolic compounds, such as flavonoids, among which isoflavones and particularly genistein are the most treated and known [91].

It remains unclear if genistein can be utilized alone or in combination with other estrogenic compounds to modulate animal reproductive function or in estrogen replacement treatment.

Genistein (7,40-dihydroxy-6-methoxyisoflavone) is commonly found in cabbage, spinach, apple, red wine, grapes, onions, tea, broccoli, strawberries, beans, and tomato [77]. In animal feed, alfalfa (*Medicago sativa*), red clover (*Trifolium pratense*), white clover (*Trifolium repens*), and soybean (*Glycine max*) are the most important plants rich in genistein and isoflavones [92]. Moreover, genistein is the most abundant isoflavone in soybeans, accounting for 60% of their total isoflavones.

The effects of flavonoids as phytoestrogens are well known in animals and women. In the latter, isoflavones and in particular genistein are used as a food supplement to relieve menopause symptoms [93]; in the latter cases, to avoid the undesirable effects of hormonal therapies (the alteration of blood coagulation with thromboembolism, coronary alterations, neoplasms (uterine, ovarian, and breast), and osteoporosis), soy-derived isoflavones are commonly used as a hormone replacement therapy [94].

In some cases, the role of genistein has also been postulated to counteract hormone-dependent cancer [84,95].

Genistein is generally perceived as a safe compound [96], but some authors recommend caution when administering high doses of genistein to menopausal women not exposed in their youth to this compound, due to the possible unknown and unpredictable risk that phytoestrogen poses on the endometrium and mammary glands [97].

Genistein activates several molecular pathways to perform these estrogenic effects. Pintova et al. [98] declare no adverse effects with genistein use. Despite this, experimental research has raised concerns about possibly harmful polyphenol overconsumption, especially for pregnant animals and their embryos, infants, and newborns, which are the most vulnerable populations in animal husbandry in this regard [99,100].

The potential of genistein as a phytoestrogen remains fairly unexplored. Several studies have reported the biochemical pathways activated by genistein and the mode of action of genistein in cell lines and animal models as well as its estrogen-like activity [77].

The molecules of genistein and its analogue daidzein resemble the structure of estradiol and many of the biological effects of isoflavonoids were initially attributed to their interactions with estrogen receptors.

Similarly to other polyphenols [101], genistein is chemically similar to 17β-estradiol, and mimics the binding of estrogens to its receptors, exerting estrogenic effects on target organs [102]. The aromatic structure of genistein contains aromatic structures able to act as estradiol A and D rings (Figure 5) [103].

**Figure 8:**
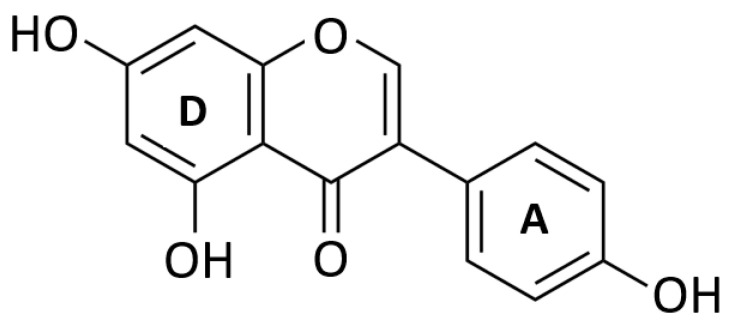
Genistein chemical structure. Genistein is characterized by aromatic structures that mimic the A and D rings of estradiol.

In 1998, Stahl [104] evidenced the estrogenic effect of genistein, zearalenone, and coumestrol on estrogen-dependent pituitary tumor cells (Figure 8).

Interestingly, genistein has a 20-fold greater affinity for ERβ than ERα; this is of particular relevance considering that the adverse effects of estrogen are due to its binding to ERα and beneficial effect on ERβ [98]. For this reason, it has been considered to be a beneficial phytoestrogen [98].

It should be noted that genistein acts on various molecular pathways to emulate the effects of estrogens.

Later, other molecular targets of isoflavonoids were discovered, including the following: topoisomerase isoform I and II, protein tyrosine kinases (v-src, Mek-4, ABL, PKC, Syk, EGFR, FGFR) directly affecting Syk kinase and anti-thrombin activity [105], ATP-binding cassette (ABC) transporters [106], ion channels (CFTR) [107], glucose transporters (Glut1, Glut4) [108,109], 5α-reductase, peroxisome proliferator-activated receptor-gamma (PPAR-γ), mitogen-activated protein kinase A, protein histidine kinase [94], and most recently, circRNA-miRNA [110]. In mouse hypothalamic GT1-7 neurons, the effect of a high dose of genistein (20 μM) on GnRH release was investigated, and it showed a significant enhancement in GnRH secretion by 122.4% in comparison to that of the control. According to the mechanistic investigation, genistein therapy may have an impact on GnRH secretion by altering the function of kisspeptin receptors, SIRT1, PKCγ, and MKRN3 in GT1-7 cells [111].

The authors confirm the role of genistein as a phytoestrogen and the aforementioned studies recommend paying attention when administering it, especially in subjects treated for the first time.

The epigenetic influence of genistein on gene expression should also be examined as emerging evidence has shown that genistein has chemopreventive action against the development of prostate and breast cancer [112].

More in detail, genistein exerts its effect using a regulatory-mediated mechanism of a nuclear receptor that can affect both estrogen [113] and androgen receptors [114]. The oral administration of genistein and estradiol affected the expression profiles of C-X-C motif chemokine ligand 12 (CXCL-12) and early growth response factor-1 (EGR-1) at both the gene and protein levels in rat ovarian cells [115].

Interestingly, higher doses of genistein (60 mg/kg) may improve ovarian function and decrease aging by altering the expression levels of CXCL-12 and EGR-1 genes and proteins in the ovary, with a synergistic impact between CXCL-12 and EGR-1 [115]. Analyzing the role of genistein as a phytoestrogen during prenatal life and its potential epigenetic effects, it is noteworthy that a differentiating tissue is more vulnerable to reprogramming than a fully differentiated tissue [116]. The effect of neonatal genistein exposure on mouse external genitalia development is a piece of evidence supporting this notion. Female mice complete urethral formation during their first few days after birth, when urethral folds emerging from urogenital sinus mesenchymal cells fuse. Female mice exposed to genistein during their first five days of life fail to complete this urethral fold union, resulting in hypospadias [117]. However, in male mice, genistein exposure during the same lifespan did not affect urethral development [117].

Several studies have shown that genistein exposure during fetal development affects DNA methylation patterns in female reproductive organs. Following neonatal diethylstilbestrol (DES) or genistein exposure, several gene promoter areas were identified to have variable methylation; one of these was Nsbp1 (now termed Hmgn5), a protein that plays a role in chromatin compaction. Following embryonic exposure to either DES or genistein, the promoter region of this gene was hypomethylated later in life (6 months of age) determining the abnormal over-expression of uterine Nsbp1 [118]. Moreover, other studies published almost two decades ago showed that neonatal exposure to DES was correlated with the hypomethylation of specific CpGs in the promoter region of the lactoferrin (Ltf) gene. Ltf is ordinarily an estrogen-responsive gene in the uterus, but genistein-induced hypomethylation has been associated with the abnormal expression of Ltf in the absence of estrogen throughout one’s life, implying that the gene’s hormone responsiveness was permanently altered [116].

Early-life xenoestrogen DES or phytoestrogen GEN exposure cause life-long reprogramming of the mouse uterine epigenome. An unbiased methylation profiling methodology was used to identify certain genes that have not been previously recognized to have relationships with the uterus [119]. These genes encode for proteins that perform a variety of biological functions.

Nucleosomal binding protein 1 (Nsbp1), a nucleosome binding and transcriptional activation element with a pivotal role in chromatin remodeling, was studied in depth as a reprogrammable gene. The findings support the hypothesis that the expression of early-life epigenetic reprogramming gene expression in the mouse uterus is dependent on adult ovarian hormones and changes during an animal’s natural aging process [119].

H3 lysine 4 trimethylation (H3K27) methylation can also be influenced by genistein exposure. The phytoestrogen genistein elevated the phosphorylation of the histone methyltransferase enhancer of zeste homolog 2 (EZH2) in neonatal rats and may have slightly reduced global H3K27me3 in the uterine myometrium [120].

Despite these promising results, further studies are necessary for a better understanding of the pathways correlated with both the phytoestrogen effect and epigenetic modulatory activity of genistein.

### 5.2. Genistein and Ovaries

Ovarian function is controlled by circulating hormones. At the hypothalamic level, estrogens, especially in the case of spontaneous ovulation, stimulate the hypothalamus to produce GnRH, which, in turn, is responsible for the release of luteinizing and follicle-stimulating hormones by adenohypophysis, causing ovulation. Phytoestrogens can interfere with this process, mimicking the action of endogenous estrogens.

Two strong pieces of evidence support the idea that an excess of phytoestrogens found in a natural diet induces decreased ovarian activity. The first is a 1940s discovery that ewes grazing in clover-rich areas in Australia had significant rates of infertility, spontaneous abortions, and reproductive abnormalities [121]. It was later discovered that the clover had large quantities of phytoestrogens [122]. A second example is a zoological population of cheetahs. These animals were infertile while being fed a soy-based diet. This diet contained high quantities of phytoestrogens and replacing it with a non-soy-based diet restored their fertility [123]. These studies show that at high enough concentrations, phytoestrogens can override natural estrogen levels. These phytoestrogens, including genistein, probably act at the hypothalamic level, suppressing ovarian activity, as drugs such as the birth control pill would.

Research published in 1998 about laboratory rodents shows the phytoestrogen-disrupting activity of genistein. This investigation found that a specific lot of unpurified soy food contained higher quantities of genistein and daidzein than ordinary batches, resulting in the estrogenic stimulation of the uterus of ovariectomized rats [124]. This yet again illustrates that phytoestrogen levels in the diet can be high enough to cause an estrogenic reaction.

A study on mice has shown that genistein has a negative impact on the developing female reproductive system. Mice who received genistein (0.5–50 mg/kg) subcutaneously on days 1–5 of their lives had altered ovarian differentiation, resulting in follicles at 2 months of age. Ovarian function and estrous cyclicity were likewise disturbed by neonatal genistein exposure, with the severity increasing over time. In mice treated with genistein (0.5, 5, or 25 mg/kg), their fertility was reduced, and infertility was found at higher doses (50 mg/kg). Neonatal genistein therapy also affected their mammary glands and behavioral outcomes. Transgenerational effects were also seen; female offspring produced by breeding genistein-treated females (25 mg/kg) to control males had higher multi-oocyte follicles [125].

Endocrine disruptor exposure definitely affects ovarian differentiation, with the outcome depending on the stage of ovarian development and exposure time. The number of female germ cells that compose the “ovarian reserve” peaks during gestation and continues to drop throughout the reproductive lifespan of female animals ([116]). The ovarian reserve is now known to be generated during gestation through a complex interplay of homeobox transcriptional factors, hormones, and genetic determinants; this process can be disturbed by environmental influences via many processes.

After birth, a variety of compounds, such as diethylstilbestrol, bisphenol A, and genistein can cause the depletion of ovarian follicle reserves because of a phytoestrogen exposure effect [126].

Genistein not only has adverse effects on the ovary and its disorders. A recent review by Nasimi Doost Azgomi (2022) summarized genistein’s effect on polycystic ovarian syndrome (PCOS) [127]. According to the findings of the different studies reported in that review [127], genistein supplementation may effectively reduce PCOS-related symptoms by lowering insulin resistance and anthropometric indicators, improving ovarian morphology and regulating reproductive hormones, and lowering oxidative stress and inflammation by influencing biological pathways. Similar results were also found by Amanat et al., 2021 [128].

Luo et al., 2020 [129] analyzed the effect of genistein on oxidative stress-mediated granulosa cell injury. They discovered that after being exposed to H_2_O_2_, genistein reduced the elevated levels of intracellular reactive oxygen species (ROS) and malondialdehyde (markers for oxidative stress) and restored the glutathione content, along with a simultaneous increase in cyclic adenosine monophosphate, whereas the addition of a protein kinase A (PKA) inhibitor stopped these effects. In this study, the oxidative stress in granulosa cells was protected by genistein via cAMP-PKA signaling.

The effect of different concentrations of genistein on LH-stimulated progesterone synthesis was tested in bovine granulosa cells. The higher genistein dosages (3700 and 3671 nmol/1) inhibited the LH-induced rise in progesterone production [130]. Similar results were obtained by Legault et al., 1999 [131] on the inhibition of progesterone production in genistein-stimulated bovine granulosa cells.

In some *in vitro* studies, genistein was used as a protein tyrosine kinase inhibitor.

In bovine luteal cells, genistein inhibited the stimulatory effects of insulin and IGF-I on thymidine incorporation [132]. Other findings in porcine oocytes show that IGF-I is at least one of the follicular stimulators of oocyte nuclear maturation, and its action is most likely not mediated by genistein-blocked tyrosine kinase-dependent intracellular pathways [133].

At 45 μM, genistein reduced the production of prolactin-stimulated progesterone below the control level in theca cells and down to the control level in luteal cells [134]. Genistein blocked the resumption of PRL-inhibited meiosis in bovine denude oocytes [135].

Genistein has different dose- and species-dependent effects in different *in vitro* cell systems [134].

As regards to IGF-I, genistein (0.001–1 µg/mL) was observed to boost IGF-I release in cultured bovine and swine granulosa cells while decreasing its secretion in rabbit granulosa cells (0.01–10 µg/mL) [136]. Regarding steroidogenesis, genistein increased progesterone secretion in rabbit and bovine granulosa cells (0.01–10 µg/mL), estradiol production in rabbit granulosa cells (1 microg/mL), and porcine ovarian follicles (10 µg/mL) [136]. Genistein (at 10 µg/mL) did not affect progesterone and PGF-2α secretion in porcine ovarian follicles. Genistein improves the cAMP production in bovine (0.001–1 µg/mL) and rabbit (at 1 µg/mL) granulosa cells [136].

In cattle, oxytocin secretion from granulosa cells is dependent on estrogens, but the nature of this process is unknown [137]. Phytoestrogens which have an affinity for the estrogen receptor may promote oxytocin secretion from bovine ovarian cells. Genistein stimulated oxytocin secretion from bovine granulosa and luteal cells in all stages of the oestrous cycle and the expression of neurophysin-I/oxytocin mRNA (this neurophysin is the precursor of oxytocin) in both types of cells [138]. As oxytocin is involved in many regulatory systems within the ovary, an increase in ovarian oxytocin production evoked by phytoestrogens throughout the estrous cycle may substantially impede reproductive functions in cows [139]. As a result, excessive oxytocin secretion may affect both premature luteolysis and the establishment of prolonged corpus lutea.

Ultimately, genistein exerts contrasting effects on ovarian function; at high doses, genistein exerts an estrogenic and antisteroidogenic effect that often suppresses ovarian activity. Beneficial effects of this isoflavone have been found in the treatment of PCOS.

### 5.3. Genistein and Pregnancy

Several scientists have described the beneficial effects of dietary/injection isoflavone genistein [140,141,142].

However, it has to be mentioned that data on its positive effect have been controversial and studies showing a completely negative effect can also be found.

Genistein (at 5 µg/mL) significantly accelerated the re-initiation and completion of nuclear maturation in pig oocytes, as well as the preimplantation development of rabbit zygotes [136].

The results of many studies show that polyphenols can affect the reproductive health of females and males and the development of embryos [75]. Also, in mouse oocytes, genistein led to a significant decrease in the rate of the following physiological processes: the rate of oocyte maturation, *in vitro* fertilization, and embryonic development. Like other food polyphenolic compounds that are part of the routine components of our diet, genistein can affect the fetal epigenome, changing it. These processes may have delayed consequences as they can be passed onto offspring through transgenerational epigenetic inheritance.

Amir et al. (2018) [143] investigated how the medium inclusion of isoflavones (genistein, formononetin, and biochanin A) can affect the outcomes of sheep oocytes. At doses of 25 µg mL^−1^, genistein decreased the cleavage rate, blastocyst rate, and blastocyst efficiency (blastocysts produced per 100 oocytes) [143]. The authors concluded that the presence of isoflavones at a concentration of 25 g mL^−1^ during IVM reduces cleavage and impairs blastocyst hatching.

Padmini and co-authors (2016) [144] studied the ability of genistein to correct oxidative stress and its effect on the placental trophoblast in the state of preeclampsia. In preeclampsia of the placental trophoblast, a significant increase in stress was observed with a decrease in the antioxidant status. At the same time, incubation with genistein significantly reduced the level of oxidative stress. Padmini reports that supplements containing genistein can be used as a mean for the prevention and treatment of preeclampsia [144].

A study in 1999 [145] shows that a background increase in estrogen levels may influence the subsequent risk of breast cancer in offspring. It was established that the exposure of the mother to the subcutaneous administration of genistein can enhance mammary tumorigenesis in her offspring, mimicking the effects of intrauterine exposure to estrogens. In addition, there may be an increase in susceptibility to carcinogen-induced mammary oncogenesis in rats exposed to genistein in the intrauterine period [145].

In 2016, Farmer [146] determined the impact of genistein when provided during late pregnancy on sows and their piglets. Injecting sows with 440 mg/day of genistein in their late gestation period increased insulin-like growth factor 1 (IGF1) concentrations in gilts and carcass fat in neonatal piglets but had a minimal effect on the muscle development of piglets at birth and on the performance of lactating sows and their litters.

To understand whether intrauterine exposure to genistein modulates postnatal respiratory allergies in middle age, a study was conducted. The use of genistein in the intrauterine period (by gavage; 20 mg/kg body weight) had a protective effect on respiratory allergies in male offspring [147].

The aim of Michikawa’s study (2019) [148] was to investigate the association between isoflavone intake during early pregnancy and hypospadias. Considering that estrogen promotes the differentiation of male external genitalia, the dietary intake of isoflavones may be associated with hypospadias, as isoflavones have a similar structure to human estrogen.

Data were used from a nationwide cohort study that included women in early pregnancy. The odds ratio of hypospadias was assessed using a logistic regression model. Michikawa and co-authors found that an increased risk of hypospadias in offspring was associated with a low maternal intake of isoflavones (genistein) in early pregnancy [148].

Based on the data obtained by Balakrishnan et al. (2010) [149], genistein can cross the placental barrier at ecologically significant levels. Placental metabolizing enzymes conjugate a small fraction of genistein into the glucuronide/sulfate form. In this form, according to the authors, genistein does not have an estrogenic effect.

Jarrell (2012) [150] remarks that women who consumed soy products had higher amniotic fluid concentrations of daidzein and genistein in pregnancies with a female fetus.

In an article by Huang (2011) [151], the effect of genistein on Lipopolysaccharide (LPS)-induced preterm birth was investigated. Their results showed that genistein can enhance the negative effect of LPS on pregnant mice by altering hormonal regulation and promoting preterm birth.

The aim of Zhang’s study (2015) [152] was to investigate the relationship between early exposure to genistein and pup body weight and to evaluate the changes in female reproductive health during puberty and adulthood after intrauterine exposure to genistein. Their high-performance liquid chromatography results showed a correlation between maternal genistein intake and its concentration in the pups’ blood plasma.

Maternal dietary supplementation with genistein has been found to reduce body weight in males and alter uterine histopathology in females [152].

In her studies (2007, 2010), Jefferson repeatedly described the negative effect of genistein on fertility. For example, mice treated neonatally with a subcutaneous injection of genistein (0.5–50 mg/kg) develop multiple oocyte follicles (MOFs). Females derived from mothers treated with genistein at 25 mg/kg crossed with normal males have elevated MOFs. The data obtained allowed scientists to assume that these negative effects can be passed onto subsequent generations [125].

The opinion of Awobajo (2022) [153] is that the mechanism of impaired fetoplacental growth, when genistein is included in the diet, can be partly explained by its interference with placental growth factor signaling.

Patel (2017) [154] states that genistein, when administered in feed, significantly reduced the duration of pregnancy in mice compared to controls. In addition, genistein reduced the litter size and increased the average pup weight.

Ultimately, the effects of genistein in pregnancy are controversial, as they depend on the species examined, the method of administration, and the doses administered.

## 6. Genistein and Male Reproductive Function

Several studies have been performed to establish the role of genistein on the male reproductive system. However, the results obtained are controversial and there is no consensus among scientists about the beneficial effect of genistein on testicular function and sperm quality [134,135,136,137,138,139,140,141,142]. This is likely due to its estrogenic effect, which actively affects testosterone synthesis.

### 6.1. Genistein in Soy Formula and Testis Function

Soy derivatives represent an important food resource for animals and humans [137]. The effects on steroidogenesis and testicular function are still being studied [133,141].

In rats, Napier and colleagues studied the effects of high doses of genistein contained in soy infant formulas on their testis function. The results obtained showed a reduction in the production of anti-Müllerian hormone by Sertoli cells. It was also shown that the soy diet stimulated the proliferation of Leydig cells during their development, while simultaneously suppressing their steroidogenic ability in adulthood [155]. However, following this study, in 2018, Applegate and colleagues published a meta-analysis based on a comprehensive updated analysis of the results obtained using genistein on male reproductive function. In this work, the authors evidenced that the consumption of soy products and their isoflavones (genistein and daidzein) is associated with a lower risk of prostate carcinogenesis. These data support the observations derived from *in vitro* and *in vivo* studies indicating that soy isoflavones deeply inhibit the development and growth of this type of male cancer [156].

Following this study, Ronis et al. (2022) stated that neither soy formula nor genistein had an estrogenic effect on neonatal pig testes. In particular, they showed that the use of soy formula during neonatal development did not have a significant effect on testis development. Moreover, other correlated studies did not determine any negative effect on the testis function following the consumption of genistein or other phytoestrogens through soy formulas during the postnatal period [157,158]. These findings were shared by several experts in the United States who further confirmed that the consumption of soy formula was a safe and cost-effective alternative to cow’s milk formula [159,160].

Further work is needed to clarify how soy-based genistein affects male reproductive function and whether this is in a dose-dependent manner.

### 6.2. Genistein and Endocrine Disruptors in Testis Function

Isoflavones, such as Genistein, exert estrogenic activity in testicular cells by direct activity on estrogen receptors (ERα and ERβ), although they have a lower affinity than estradiol [161]. The assumption that these phytoestrogens regulate the synthesis of testosterone is demonstrated by the high amount of testosterone in the serum of male mice deficient in the ERα gene [162]. Leydig cells synthetize testosterone, which is the primary form of male steroid hormones. In particular, the enzyme Cytochrome P45017A1 (CYP17A1) present in Leydig cells catalyzes testosterone synthesis [163]. Benson and colleagues showed that the deficiency of the ERα receptor, due to the deletion of the ERα gene (αERKO), was correlated with increased testosterone biosynthesis. In the same study, the authors determined that the amount of serum testosterone and the transcript levels of several steroidogenic enzymes, such as CYP17A1, were significantly higher in adult αERKO mice compared with those of wild-type mice [164]. These findings show that isoflavones can disrupt ERα receptors determining testosterone synthesis disorder.

Several studies have demonstrated that genistein has an antioxidant effect [165,166,167]. In particular, Zhao and colleagues demonstrated that this phytoestrogen mitigates the negative effects of phthalates, which are well-known endocrine disruptors. In this study, the authors showed that low doses of mono-(2-ethylhexyl) phthalate (MEHP) caused oxidative stress damage, inhibiting the development of the testis and the development of Sertoli and Leydig cells during pregnancy. The administration of genistein to pregnant females exposed to MEHP attenuated the negative impact of these endocrine disruptors on fetal testicular development. This seemed to be exerted through genistein antioxidant effects as indicated by assessments of testicular cell markers, the testosterone concentration, the redox state of tissues, and a morphological analysis of the testicular parenchyma. The authors postulated that the consumption of isoflavones diminished the fetal testes’ susceptibility to damage following exposure to phthalates [166].

In agreement with these results, Zhang and colleagues parallelly administrated di-(2-ethylhexyl) phthalate (DEHP) and genistein at different doses to male prepubertal rats. The results obtained showed that genistein partially mitigated the damage to the testicular tissue caused by DEHP and increased the activity of testicular antioxidant enzymes as shown by the testis weight, anogenital distance, and organ ratio. Once again, it was demonstrated that genistein reduced the disruptive effects of endocrine factors correlated with reproductive disorders [168].

In 2020, in rats, Walker and colleagues evaluated the exposure effects of 0.1 and 10 mg/kg/day of GEN and DEHP on testicular function during pregnancy and at birth. Their results indicate that the combination of GEN and di(2-ethylhexyl) phthalate (DEHP), at doses to which humans can be physiologically exposed, induced alterations in the morphology of the testis. Moreover, a transcriptome analysis revealed that the expression of several transcripts was altered exclusively by the mixtures than by the individual compounds, suggesting simultaneous age-dependent and non-monotomic changes. In the same study, the combination of GEN and DEHP increased the number of innate immune cells, such as macrophages, indicating inflammatory responses that may contribute to gonadal dysfunction [169]. Recently, the same authors examined the effect of genistein on the germ cells of adult rats after prenatal exposure using doses typical of human diets. The results of this study indicated that, in utero, low doses of genistein together with DEHP exposure may disrupt the development of testicular cells, including Leydig cells [170]. Recently, in another study, acetaminophen (APAP) and genistein were used individually or in combination to evaluate their effect on two *in vitro* models of immature Sertoli cells: mouse Sertoli TM4 cells and postnatal day 8 (PND8) rat Sertoli cells.

The results obtained indicated that the exposure of both cell lines to APAP and GEN alone and as mixtures dysregulated cell function and development. Moreover, a gene expression analysis evidenced similar effects of APAP and GEN on critical genes and biological functions, including on Cox-related genes.

In conclusion, these findings indicate a possible interaction between genistein and endocrine disruptors. Therefore, in future studies, it is necessary to better understand the reason underlying this phenomenon.

### 6.3. Genistein and Sperm Quality

The beneficial effect of genistein on sperm quality is still being debated among scientists, although several studies have been performed [171,172]. In a recent work by Yao et al., it was established that sperm treatment with genistein for 1 h at 27 °C reduced the sperm DNA fragmentation of bull sperm, although the pronuclear formation rate after *in vitro* fertilization (IVF) and sperm motility were reduced. This result could be interesting when using sperm samples with greater sperm DNA fragmentation and when motility variables are not as important as those for ICSI [173]. However, several studies have indicated a clear negative effect of genistein on sperm quality due to, among others, alterations in the ultrastructure of the testes with consequences on male fertility [174,175,176,177].

In another study on rabbits, the beneficial effect of genistein was observed only for a brief period, whereas prolonged exposure to this phytoestrogen led to the alteration of sperm quality. In this study, diets containing high concentrations of isoflavones or lignans did not influence the reproductive performance of bucks. However, in the first period of the feeding, the sperm number, motility, and functionality increased in the bucks fed a diet containing phytoestrogen. However, after prolonged exposure, a reduction in sperm concentration and increased sperm morphology were observed [178].

Recently, Caceres and colleagues examined the effects of prolonged exposure to isoflavones on testicular function in adult male rats administrated mixtures with varying levels of isoflavones (genistein and daidzein) for 5 months. The results obtained showed that isoflavones induced alterations in the synthesis of androgens and estrogens, resulting in decreased levels of circulating testicular androgens and increased levels of estrogen. This, in turn, determined a decrease in the sperm quality, testicular weight, diameter of the seminiferous tubules, and height of the germinal epithelium [174].

Overall, these findings clearly show that the beneficial effect of genistein is dependent on the dose, the species, and the time of administration.

## 7. Conclusions

Genistein is a flavonoid with antioxidant, anti-inflammatory, and anti-neoplastic properties. Different studies have found that genistein influences both female and male reproduction.

Genistein is classified as a phytoestrogen in females, with a chemical structure comparable to that of certain estrogens. As a result, it can bind to Erα and Erβ receptors, with a preference for the latter, regulating several pathway molecules such as the following: topoisomerase isoform I and II, protein tyrosine kinases (v-src, Mek-4, ABL, PKC, Syk, EGFR, FGFR), ABC, CFTR, Glut1, Glut4, 5α-reductase, PPAR-γ, mitogen-activated protein kinase A, protein histidine kinase, and most recently, circRNA-miRNA. Genistein may be useful alone or in conjunction with other hormones due to its modest estrogenic activity. In females, genistein has opposing effects on ovarian function; at high doses, genistein has estrogenic and anti-steroidogenic activity, which frequently lowers ovarian activity. This isoflavone has been reported to have beneficial effects on the treatment of PCOS. Finally, the effects of genistein on pregnancy are debatable because they are dependent on the species studied, the route of administration, and the amounts used.

In males, genistein appears to have an estrogenic stimulating action and interferes with testis function by interacting with both natural and synthetic endocrine disruptors. The effect of genistein on sperm quality varies based on the dose, the species, and the time of administration.

Genistein has potentially adverse epigenetic effects in the female and male reproductive tract. However, further investigations are needed on this subject.

In conclusion, despite evidence indicating an impact of genistein on human and animal reproduction, further studies are needed to consolidate our knowledge on the characteristics of genistein and its role in mammalian reproductive function.
